# Impact of lead detecting algorithms on inappropriate shocks in implantable cardioverter defibrillator lead failure: a single-center manufacturer-independent observational study

**DOI:** 10.1007/s10840-022-01460-1

**Published:** 2022-12-29

**Authors:** Eric Lemmermöhle, Korbinian Lackermair, Ina Klier, Sebastian Sadoni, Oliver Heyn, Bonnie Hartrampf, Valentina Seitelberger, Thomas Czermak, Antonia Kellnar, Moritz Sinner, Heidi Estner, Stephanie Fichtner

**Affiliations:** 1 Department of Cardiology, Munich University Hospital, Ludwig-Maximilians-UniversitätMünchen (LMU Munich), Munich, Germany; 2 Department of Cardiac Surgery, Munich University Hospital, Ludwig-Maximilians-Universität München (LMU Munich), Munich, Germany

## Introduction

Implantable Cardioverter Defibrillators (ICDs) are well-proven in preventing sudden cardiac death in patients who are at risk for life-threatening ventricular arrhythmia [1]. Major challenges that accompany the widespread use of ICDs are high up-front costs and device-related complications [2]. Lead failure is a relevant complication with long-term failure rates ranging from 10 to 40% [3]. This frequently leads to inappropriate arrhythmia detection with consecutive shock delivery and potential severe impact on physical and mental health [4]. Many of the more advanced ICD models incorporate diagnostic algorithms, that have previously shown evidence in detecting lead failure early and reducing inappropriate shocks [5]. Remote monitoring (RM) of ICDs is another diagnostic tool that couples with lead failure algorithms and proved to be effective in reducing inappropriate therapies [6]. In order to lower the high up-front costs of ICDs, many centers still tend to implant simpler devices without lead alert function, as well as not implementing RM which takes up additional resources [7]. The following study was initiated to investigate the distribution and effect of lead failure diagnostics in a manufacturer-independent real-world setting.

## Methods

### Study design

The present single-center, observational study was conducted at the Munich University Hospital, Ludwig-Maximilians-Universität München (LMU Munich). The study protocol followed the principles of the Declaration of Helsinki and was approved by the institutional ethics committee. Informed consent was waived given the retrospective nature of this study.

### Study population and data collection

All consecutive patients with clinical diagnosis of ICD lead failure and following surgical lead revision from January 1st, 2013 to December 31st, 2019 were retrospectively analyzed. Patient information and lead data were extracted from electronic medical records in the context of standard practice. Patients with lead dislodgement and lead revision due to an infected device system were excluded.

### Definitions and lead failure detection

Lead failure was detected from routine clinical visits with subsequent ICD interrogation or urgent presentation due to inadequate ICD therapy or lead alert. Diagnosis was made on the basis of clinical failure manifestation, including abnormalities in sensing, pacing and impedance. Lead failure and consecutive indication for surgical revision were reviewed and adjudicated by at least two physicians with at least one being an interventional cardiologist or cardiac surgeon familiar with lead extraction procedures. The following lead failure algorithms were included in this study: Medtronic Lead Integrity Alert, Medtronic Lead Noise Algorithm, Boston Scientific Latitude Lead Alert, Abbott SecureSense RV Lead Noise Discrimination. Remote monitoring platforms included the Medtronic CareLink Network, Biotronik Home Monitoring, Boston Scientific Latitude Remote Patient Management System, and Abbott Merlin Patient Care Network. Algorithm and remote monitoring design has been previously described.

### Statistical analysis

Quantitative variables were presented as median and compared using the Mann–Whitney *U* test. Qualitative variables were expressed as frequencies and compared via chi-squared or Fisher’s exact test. All *P* values were two-tailed. *P* values < 0.05 were considered to be statistically significant. All statistical analysis was performed using IBM SPSS, Version 26 (IBM Inc., Armonk, NY, USA).

## Results

### Patient and lead failure characteristics

**Fig. 1:**
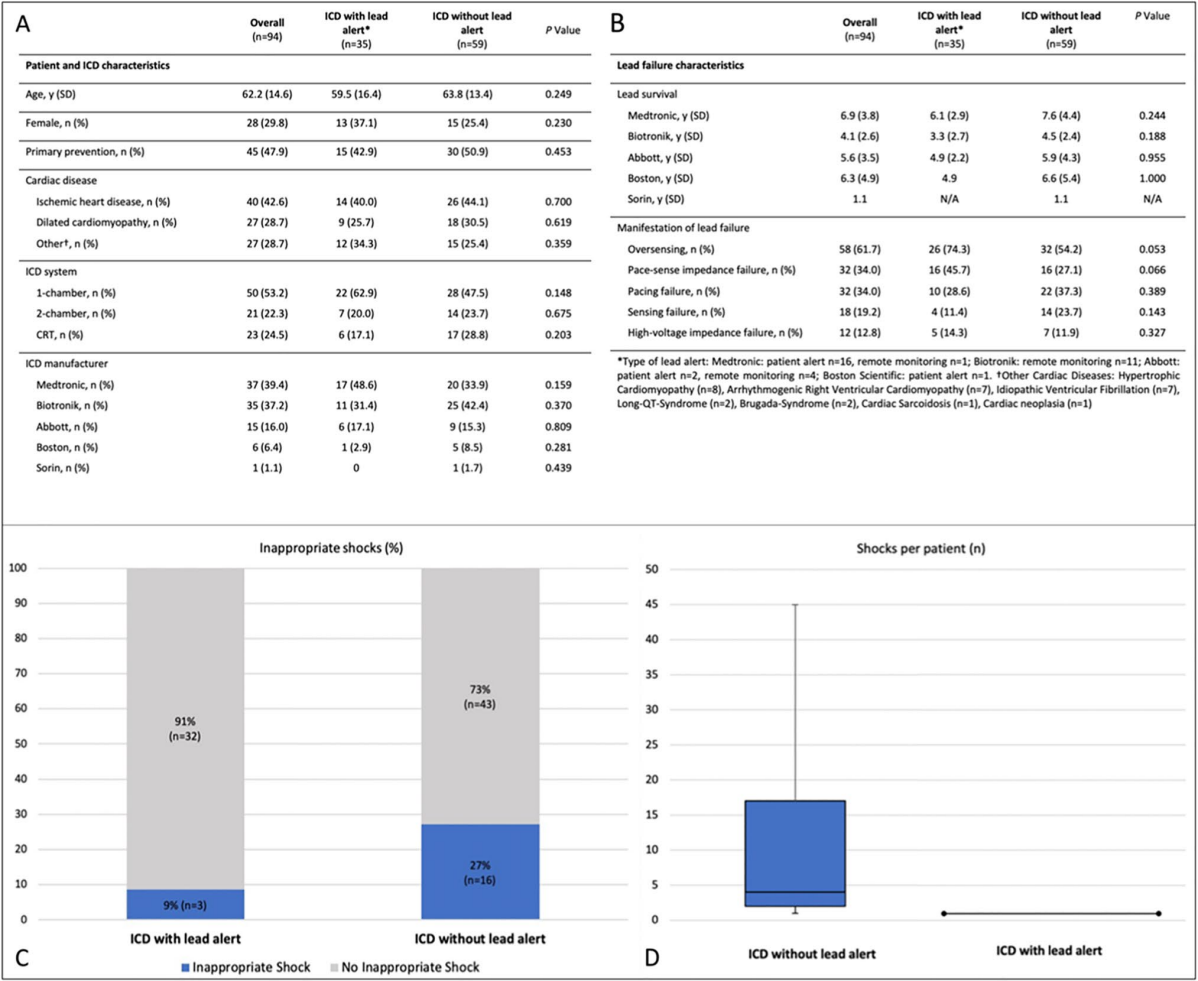
**A**,** B** Comparison of patient, ICD, and lead failure characteristics based on lead failure detection. **C**,** D** Incidence and frequency of inappropriate shocks based on lead failure detection

Overall, 94 consecutive patients with ICD lead failure and no evidence of lead dislodgement or infected ICD system were included. Baseline and lead failure characteristics are summarized in the Fig. 1A, B. The study population was divided into two groups depending on lead failure detection. Detection through lead failure algorithms (*n* = 19) or remote monitoring (*n* = 16) was present in 35 of 94 patients (37%), while 59 of 94 patients (63%) had conventional follow-up in the context of routine or urgent device interrogation.

### Incidence and frequency of inappropriate shocks

Overall, 19 patients (20%) experienced inappropriate shock therapy with an average of 9.3 shocks per patient. Comparison of the groups shows that patients with lead alert had a significantly lower risk of receiving inappropriate defibrillation (Fig. 1C). With lead alert 3 out of 35 patients (9%) suffered from inappropriate shocks versus 16 out 59 patients (27%) without lead alert (*P* = 0.03). Furthermore, in cases without lead alert there was a significant higher chance of receiving multiple shocks with an average of 11.0 shocks per patient compared to 1.0 shock per patient in the lead alert group (Fig. 1D; *P* = 0.028).

## Discussion

The following study demonstrated a high overall incidence of inappropriate shocks in ICD lead failure with every fifth patient being affected. ICDs with detecting algorithms for lead failure or remote monitoring alert had a significantly lower incidence and frequency of inappropriate shocks. Without lead alert there was a threefold higher risk for inappropriate defibrillation, as well as a much greater chance of receiving multiple shocks. Considering relatively high long-term ICD lead failure rates with an associated risk of inappropriate defibrillation and potential severe harm on mental and physical health, implantation of ICDs without lead alert function has to be viewed highly critical. Several limitations need to be considered including the small sample size and retrospective observational nature of this study. Further analysis including by manufacturer and device settings were limited by sample size, implantation practice at our center and research letter format.

## Conclusions

Our study provides evidence that lead failure detecting algorithms and remote monitoring significantly reduce inappropriate shocks in ICD lead failure. Given the well-known high long-term failure rate of ICD leads, more advanced ICDs with device-based lead failure algorithms should be used to reduce harmful inappropriate shocks, even though they are more expensive upfront.

## Data Availability

Data used in this study is available from the corresponding author upon request.
